# The Impact of 3D Interactive Prompts on College Students’ Learning Outcomes in Desktop Virtual Learning Environments: A Study Based on Eye-Tracking Experiments

**DOI:** 10.3390/jemr19010019

**Published:** 2026-02-05

**Authors:** Xinyi Wu, Xiangen Wu, Weixing Hu, Jian Sun

**Affiliations:** 1 College of Education, Dalian University, Dalian 116622, China; wuxy558@nenu.edu.cn; 2 School of Educational Science, Shenyang Normal University, Shenyang110034, China; wuxiangen@gmail.com; 3 School of Music and Dance, Xihua University, Chengdu 610039, China; 0120210048@mail.xhu.edu.cn

**Keywords:** eye-tracking, desktop virtual learning environment (DVLE), 3D interactive prompts, learning outcomes

## Abstract

Despite the increasing adoption of desktop virtual reality (VR) in higher education, the specific instructional efficacy of 3D interactive prompts remains inadequately understood. This study examines how such prompts—specifically dynamic spatial annotations and 3D animated demonstrations—influence learning outcomes within a desktop virtual learning environment (DVLE). Employing a quasi-experimental design integrated with eye-tracking and multimodal learning analytics, university students were assigned to either an experimental group (DVLE with 3D prompts) or a control group (basic DVLE) while completing physics tasks. Data collection encompassed eye-tracking metrics (fixation heatmaps, pupil diameter and dwell time), post-test performance (assessing knowledge comprehension and spatial problem-solving), and cognitive load ratings. Results indicated that the experimental group achieved significantly superior learning outcomes, particularly in spatial understanding and dynamic reasoning, alongside optimized visual attention patterns—characterized by shorter initial fixation latency and prolonged fixation on key 3D elements—and reduced cognitive load. Eye-tracking metrics were positively correlated with post-test scores, confirming that 3D prompts enhance learning by improving spatial attention guidance. These findings demonstrate that embedding 3D interactive prompts in DVLEs effectively directs visual attention, alleviates cognitive burden, and improves learning efficiency, offering valuable implications for the design of immersive educational settings.

## 1. Introduction

Desktop Virtual Learning Environments (DVLEs) have emerged as essential tools in higher education, offering immersive, flexible, and often cost-effective learning alternatives or supplements to traditional instructional modalities [1,2]. These environments are designed to simulate complex scenarios, visualize abstract concepts, and enable experiential learning that would otherwise be difficult, risky, or impossible under real-world constraints. Consequently, DVLEs have been shown to hold significant potential for enhancing students’ engagement with and comprehension of disciplinary content across diverse fields [3,4]. However, the full instructional potential of DVLEs has not yet been adequately realized. Learners may experience excessive cognitive load, disorientation, or passivity within these complex three-dimensional spaces, thereby hindering knowledge acquisition and deeper cognitive processing [5,6]. Without adequate guidance, students may struggle to identify key information, comprehend spatial relationships, or apply effective cognitive strategies [7]. Consequently, the development of effective scaffolding mechanisms within DVLEs has become increasingly urgent, as they would allow learners’ attention to be directed, active processing to be facilitated, and self-regulated learning to be supported and enhanced [8,9].

Interactive prompts have been identified as a promising scaffolding strategy. These prompts are strategically embedded as cues, questions, directives, or feedback mechanisms with the aim of evoking specific cognitive or behavioral responses from learners [10,11]. Within three-dimensional Desktop Virtual Learning Environments (DVLEs), prompts can play various roles, including highlighting key objects, posing guiding questions, suggesting exploration paths, eliciting reflection, or providing metacognitive support. From a theoretical standpoint, well-designed prompts are understood to alleviate extraneous cognitive load, direct learners’ attention to essential information (i.e., germane cognitive load), and facilitate generative processing, thereby enhancing learning outcomes [5,9,12]. Although research into multimedia learning has demonstrated the effectiveness of prompts in two-dimensional learning environments [13], their specific impact on university students’ learning performance within the structurally more complex and highly interactive three-dimensional DVLEs remains underexplored.

A clear understanding of how learners process three-dimensional interactive prompts is essential for the optimization of their design. Traditional measures of learning outcomes, such as retention and transfer tests, offer valuable summative data but provide only limited insights into learners’ underlying attentional allocation and cognitive activities during the learning process [14]. Eye-tracking technology provides an efficient and non-invasive method for the real-time capture of these cognitive processes. By recording indicators such as gaze position, fixation duration, saccades, and scan paths, eye tracking enables objective assessment of learners’ visual attention distribution, information-selection processes, and levels of cognitive engagement [15]. Prior research on multimedia learning has employed eye tracking to reveal how cueing signals guide learners’ attention [7,16] and to identify relationships between specific attentional patterns and learning outcomes [17]. Applying eye-tracking methods to investigate interactive prompts within three-dimensional Desktop Virtual Learning Environments (DVLEs) enables researchers to move beyond correlational analyses and examine in greater depth the mediating role of attentional processes. Such an approach may clarify, for example, whether prompts effectively direct learners toward essential information, whether visual-attention patterns can serve as predictors of subsequent learning outcomes, and how prompt design influences the level of cognitive engagement inferred from eye-movement data. Addressing these questions is essential for the refinement of prompt design in DVLEs.

Despite the theoretical significance and practical potential of three-dimensional interactive prompts, along with the unique analytical perspective afforded by eye-tracking technology, a notable gap persists in the existing literature. Relatively few studies have systematically examined the specific effects of different types of three-dimensional interactive prompts on university students’ learning outcomes in Desktop Virtual Learning Environments (DVLEs), and even fewer have integrated eye-tracking methods to uncover the attentional mechanisms underlying such prompts [18]. Addressing this gap is essential for establishing empirically grounded design principles and for providing effective instructional scaffolding within immersive educational technologies. University Physics (also referred to as General Physics) is a foundational first-year course for students in science and engineering, characterized by the introduction of calculus-based reasoning that deepens understanding of high-school physics concepts and moves instruction beyond the rote application of formulas. The course typically encompasses mechanics, electromagnetism, thermodynamics, and optics. In the context of mechanics instruction, traditional lecture-based approaches have been insufficient in providing students with a truly immersive and interactive learning environment. To address these challenges, the present study integrates three-dimensional interactive prompts with eye-tracking technology to construct an immersive DVLE with the aim of examining their influence on university students’ conceptual understanding and problem-solving performance in mechanics. By comparing how different types of prompts influence attention allocation, cognitive load, and learning outcomes, the study further elucidates the ways in which visual guidance and interaction design shape students’ learning trajectories in University Physics.

The present study was designed to address this gap by investigating the effects of three-dimensional interactive prompts in Desktop Virtual Learning Environments (DVLEs) on university students’ learning outcomes and by employing eye-tracking technology as the primary methodological tool to uncover the attentional processes underlying these effects. Specifically, the study was guided by the following research questions:

1. How are university students’ knowledge acquisition, comprehension, and transfer abilities affected by different types of 3D interactive prompts?
2. In what ways do these prompts shape learners’ behaviors in DVLEs through the visual-attention allocation and cognitive-processing patterns reflected in eye-tracking indicators?
3. What are the relationships between the visual-attention patterns elicited by the prompts and subsequent learning outcomes?

## 2. Literature Review

### 2.1. The Rise of Desktop Virtual Learning Environments and Associated Instructional Challenges

Desktop Virtual Learning Environments (DVLEs), with their strong visual and interactive capabilities, have been shown to offer distinct advantages in supporting the understanding of abstract concepts such as physical mechanics and molecular structures. A growing body of research has demonstrated that DVLEs can effectively enhance students’ learning motivation and foster contextualized cognition [19]. However, meta-analytic findings indicate that the learning benefits of DVLEs vary considerably and depend primarily on instructional design rather than the technology itself [20]. Among the challenges identified, cognitive load management has been identified as particularly critical. Although the rich visual information and open-ended exploration typically afforded by DVLEs can stimulate learner interest, they may also lead to a substantial increase in extraneous forms of cognitive load. Learners unfamiliar with navigating three-dimensional interfaces or interpreting complex scenes may struggle to efficiently identify relevant information, resulting in the diversion of cognitive resources that should support knowledge construction. Consequently, for learners with limited prior knowledge, minimally guided “exploratory” DVLEs may produce counterproductive outcomes.

### 2.2. Designing Support in Desktop Virtual Learning Environments: From Two-Dimensional Cues to Three-Dimensional Interactive Prompts

To address these challenges, the integration of instructional support within learning environments has been widely recognized as essential. In multimedia learning research, signaling techniques—such as arrows or color highlighting—have been shown to effectively guide learners’ visual attention and to facilitate the integration of key information [21]. However, traditional two-dimensional cues exhibit inherent limitations in three-dimensional environments: they are typically overlaid onto the screen plane and spatially separated from three-dimensional objects, a mismatch that may weaken their guiding function or introduce cognitive conflict.

In recent years, increasing attention has been directed toward interactive prompts that are natively embedded within three-dimensional space. These prompts are directly linked to objects or operational steps within the environment and commonly take the form of:

(1) Dynamic spatial annotations, in which text labels or icon-based indicators appear adjacent to three-dimensional objects and continue to indicate the target as the learner’s viewpoint shifts, thereby reducing visual search demands [22]; and
(2) Three-dimensional animated demonstrations, which visually present physical processes or procedural actions—such as force decomposition or mechanical operations—to make abstract concepts more readily accessible and support the construction of dynamic mental models [23].

The design of such three-dimensional interactive prompts draws upon key principles of Cognitive Load Theory and of the Cognitive Theory of Multimedia Learning [9]. By optimizing information presentation (signaling principle), directing learner attention to relevant content (attentional-guidance principle), and reducing the working memory burden associated with mental simulation through the visualization of complex processes (spatial–temporal contiguity principle), these prompts effectively manage learners’ cognitive resources and facilitate schema construction.

### 2.3. Eye-Tracking and Multimodal Learning Analytics: A Window into the Learning Process

Although three-dimensional interactive prompts hold considerable theoretical advantages, research designs based solely on pre- and post-test comparisons are insufficient for elucidating their underlying mechanisms of action. Key questions remain unresolved, including whether learners allocate attention in the manner intended by instructional designers and how cognitive load fluctuates dynamically throughout the learning process. Addressing such issues requires methodological tools capable of capturing learning processes at a fine level of granularity, and eye-tracking technology has been recognized as an effective solution in this regard. In recent years, multiple eye-movement indicators have been used to objectively quantify learners’ visual–cognitive processing within Desktop Virtual Learning Environments (DVLEs). These indicators include first-fixation duration (an indicator of information-access efficiency), fixation heatmaps (reflecting patterns of attentional distribution), pupil diameter (an indirect physiological marker of cognitive load), and spatial fixation depth (distinguishing between foreground and background attentional behavior within three-dimensional scenes) [24]. Empirical evidence has shown that effective instructional prompts significantly reduce learners’ first-fixation times on key information and increase the total fixation duration within core regions. Multimodal learning analytics—integrating eye-tracking data with performance measures and subjective cognitive-load ratings—has increasingly emerged as a leading research paradigm. Such combined analyses help establish causal chains linking “instructional intervention–process behavior–learning outcomes,” thereby providing empirical support for explaining the mechanisms through which specific design strategies are effective [25]. Taken together, existing studies have preliminarily confirmed the need for instructional support in DVLEs and have begun to explore the potential value of three-dimensional interactive prompts. However, several substantial gaps remain: (1) Unclear mechanisms: current research rarely offers process-based evidence showing how 3D prompts guide visual-attention pathways, influence cognitive processing, or reduce cognitive load; (2) Methodological limitations: reliance on questionnaires and test scores limits the ability to capture real-time attentional allocation or moment-to-moment fluctuations in cognitive load; and (3) Ambiguous design principles: empirically grounded guidance regarding which types of 3D prompts are most effective under specific learning conditions has yet to be clearly established.

Accordingly, this study adopted a quasi-experimental design that integrated eye-tracking technology with multimodal learning-analytics methods to systematically examine the effects of three-dimensional interactive prompts—specifically dynamic spatial annotations and three-dimensional animated demonstrations—on university students’ learning outcomes, visual-attention patterns, and cognitive load during physics-learning tasks. The study aimed not only to validate the instructional effectiveness of 3D interactive prompts but also to elucidate their underlying mechanisms through detailed process data. In doing so, it sought to provide a stronger theoretical foundation and to refine the design principles necessary for constructing immersive learning environments.

## 3. Materials and Methods

### 3.1. Methods

#### 3.1.1. Experimental Design

A 2 × 2 between-subjects quasi-experimental design was employed, which incorporated independent, dependent, and controlled variables. The independent variables were the type of interactive prompts embedded in the Desktop Virtual Learning Environment (DVLE)—either conventional interactive prompts or 3D interactive prompts—and the difficulty level of the learning content. The introductory level included common forces in Newton’s laws of motion (gravity, elastic force, and friction), whereas the intermediate level focused on the rotational form of Newton’s second law. The dependent variables consisted of measures of learning performance (retention and transfer test scores), eye-tracking indicators (fixation duration, fixation count, and saccade count), and cognitive load. Prior knowledge differences among participants were controlled through random assignment to experimental conditions. All participants were first-year university students enrolled in the same introductory physics course, minimizing systematic differences in academic background.

#### 3.1.2. Participants

The participants were first-year undergraduates enrolled in a university physics course at an experimental university located in L Province of Country C. A total of 232 students were selected through random sampling and were subsequently randomly assigned to four groups: Experimental Group 1 (*n* = 58), Experimental Group 2 (*n* = 58), Control Group 1 (*n* = 58), and Control Group 2 (*n* = 58). The gender distribution was balanced across all groups. All participants had normal or corrected-to-normal vision, reported no color blindness or color weakness, and were otherwise in good physical health. None had prior experience with Desktop Virtual Learning Environments (DVLEs). Participation was voluntary, and all students provided written informed consent. The study protocol was reviewed and approved by the Academic Ethics Committee of Xihua University (Approval No. 2025-K001).

#### 3.1.3. Eye-Tracking Apparatus & Metrics

**Figure 1:**
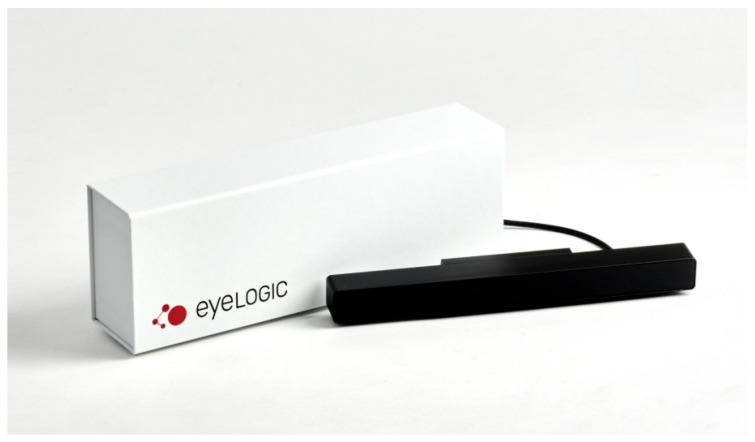
Schematic diagram of the Eye Logic One eye tracker.

As shown in Figure 1, eye-movement data were collected with the Eye Logic One eye tracker manufactured by Eye Logic (Berlin, Germany). The device is based on a three-dimensional model of the human eye and employs a remote gaze-tracking algorithm that is capable of accurately computing gaze coordinates within a range of 55 cm to 80 cm without requiring head stabilization. This non-contact, head-free measurement approach provided participants with a naturalistic and comfortable experimental experience.

#### 3.1.4. Assessment Indicators and Instruments

(1) Eye-Tracking Indicators

Eye-tracking metrics provide real-time, objective indicators of cognitive processing, thereby enabling the investigation of learners’ attentional allocation, processing efficiency, and cognitive strategies. In this study, fixation duration, fixation count, and saccade count were used as the primary indicators and were supplemented with fixation heatmaps and scan-path visualizations in the experimental groups to support integrated analyses. Fixation duration is defined as the time during which the eye remains stationary within a designated area of interest (AOI), reflecting both the depth of cognitive processing and the extent of cognitive effort devoted to specific information. Fixation count is defined as the total number of fixations recorded within an AOI and serves as an indicator of the intensity of attention directed toward that region and the efficiency of visual search. Saccade count is defined as the number of rapid eye movements occurring between successive fixations and serves as a behavioral marker of attentional shifts. Collectively, these eye-tracking indicators characterize learners’ visual-behavior patterns (“how they look”) and elucidate the cognitive processes underlying their learning (“how they think”). Eye-movement data complement performance outcomes and offer process-level evidence that helps explain the mechanisms through which different instructional methods generate differential learning effects.

(2) Learning Performance

Learning performance serves as a primary indicator of students’ mastery of university-level physics concepts. In this study, both retention and transfer tests were employed, with all items developed by the university’s physics teaching team. The retention test was designed to assess students’ memory and comprehension of the learned content—namely, their direct grasp of instructional materials—using test items tightly aligned with the instructional content. In contrast, the transfer test was designed to evaluate students’ ability to apply acquired knowledge to novel contexts and problem scenarios, a process that involves higher-order cognitive abilities such as analysis, synthesis, and creativity. By combining retention and transfer assessments, the study captured the full trajectory from “knowledge retention” to “knowledge application,” thereby offering a more comprehensive evaluation of learning outcomes than could be achieved through a single performance measure.

(3) Cognitive Load

Cognitive load was assessed using a standardized subjective cognitive-load rating scale. The scale provides a subjective assessment of learners’ perceived mental effort and task difficulty, thereby capturing their cognitive experience throughout the learning process. In this study, the subjective cognitive-load rating scale developed by Paas and van Merriënboer was utilized. The scale demonstrated good internal consistency, with a Cronbach’s alpha of 0.79.

Although pupil diameter and spatial fixation depth were recorded during the experiment, these metrics were excluded from the primary analysis due to high measurement variability under ambient lighting conditions and the focus of this study on attentional allocation rather than perceptual depth processing.

#### 3.1.5. Procedure

The pre-experimental phase (approximately 10 min) included an overview of the experimental procedure, followed by the completion of a demographic questionnaire and a physics proficiency pre-test. Subsequently, calibration and adjustment of the screen-based eye tracker were performed.

During the experimental phase (15–25 min), participants completed their assigned physics-learning tasks in accordance with group conditions. In the groups lacking three-dimensional interactive prompts, the learning environment excluded dynamic spatial annotations and 3D demonstrations, and the average task completion time was approximately 25 min. In the experimental groups, students engaged in five to eight interactive operations, all involving dynamic spatial annotations or 3D demonstrations; in the control groups, an equivalent number of interactions was supported by conventional prompts. Based on the difficulty of the physics content, tasks were classified into introductory and intermediate levels. Accordingly, the four experimental conditions were defined as follows: introductory-level DVLE with conventional prompts, introductory-level DVLE with 3D interactive prompts, intermediate-level DVLE with conventional prompts, and intermediate-level DVLE with 3D interactive prompts.

#### 3.1.6. Materials

**Figure 2:**
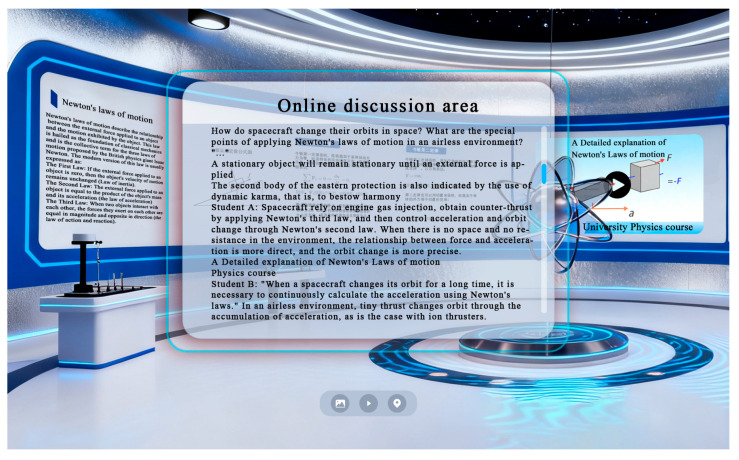
Desktop-based virtual physics learning environment.

As shown in Figure 2, the experimental materials comprised a desktop-based virtual physics learning environment developed in-house by the research team. The platform was constructed through 3D rendering of instructional content using 3ds Max 2025 and implemented in the Unity engine. The design of the virtual learning content strictly adhered to the instructional standards of university physics courses and was designed to provide an immersive, high-quality interactive learning environment. The materials displayed in Figure 2 correspond to those used in the experimental groups and feature both 3D interactive prompts and 3D demonstrations. The learning content included introductory materials on Newton’s first law and intermediate materials on Newton’s second law.

#### 3.1.7. 3D Interactive Prompts Description

The 3D interactive prompts used under the experimental conditions consisted of two primary forms:

(1) Dynamic spatial annotations: textual labels and directional arrows anchored to 3D objects (e.g., force vectors, pivot points) that remained spatially aligned with the object as the viewpoint changed.
(2) 3D animated demonstrations: short, auto-played animations illustrating physical processes (e.g., torque application, angular momentum conservation) within the virtual scene.

### 3.2. Data Analysis

All statistical analyses were conducted using SPSS 27 and RStudio 2024.012.1+563. The significance level for all inferential tests was set at α = 0.05. The analytical procedure consisted of the following stages:

1. Descriptive Statistics:

Means and standard deviations were computed for learning performance (retention and transfer test scores), eye-tracking indicators (fixation duration, fixation count, and saccade count), and cognitive load, and their distributional characteristics were examined.

2. Comparative Analysis:

To compare the effects of the two interactive prompt types and the two levels of task difficulty on learning performance, eye-movement indicators, and cognitive load, two-way analyses of variance were performed on datasets that met the assumption of homogeneity of variance.

3. Interaction Analysis:

Analyses of variance combined with visualization techniques were used to examine how interactive prompt type and task difficulty jointly influenced learning performance, eye-tracking measures, and cognitive-load levels.

4. Visualization:

A series of visual representations of the results were generated, including heatmaps illustrating gaze patterns in the experimental groups, faceted density plots depicting the distribution of collected data, interaction plots displaying the joint effects of prompt type and task difficulty, and correlation matrices showing the strength of associations among variables.

## 4. Results

### 4.1. Descriptive Statistical Analysis

Based on the data presented in Table 1, the combined effects of interactive prompt type and task difficulty on each variable can be systematically evaluated. In terms of academic performance, both retention and transfer test scores were higher under the 3D interactive condition than under the conventional condition, with the highest scores recorded at the intermediate difficulty level. When 3D interaction was paired with intermediate-level content, the mean retention score reached 82.28, and the mean transfer score reached 84.26, constituting the highest values across all groups. Notably, under the conventional interaction conditions, performance at the introductory level exceeded that at the intermediate level, indicating that traditional interaction formats may be insufficient to support more complex learning content.

For the eye-tracking indicators, fixation duration, fixation count, and saccade count were consistently higher under the 3D interactive condition than under the conventional format, with the 3D-interaction/intermediate-difficulty combination yielding the highest values. For example, the mean fixation count reached 132.53 and the mean saccade count reached 86.02, suggesting more active information processing, enhanced visual search, and greater cognitive engagement. From the perspective of cognitive load, overall cognitive-load levels were lower under the 3D interactive condition, with the lowest mean value (1.74) observed in the 3D-interaction/intermediate-difficulty condition, whereas the highest level (mean = 3.14) occurred under the conventional-interaction/intermediate-difficulty condition. These findings indicate that 3D interaction can mitigate the cognitive demands imposed by more challenging content and improve learners’ psychological comfort during learning. Additionally, the absolute values of skewness and kurtosis for all variables were below 3 and 10, respectively, indicating approximate normality and satisfying the assumptions required for parametric testing.

**Figure 3:**
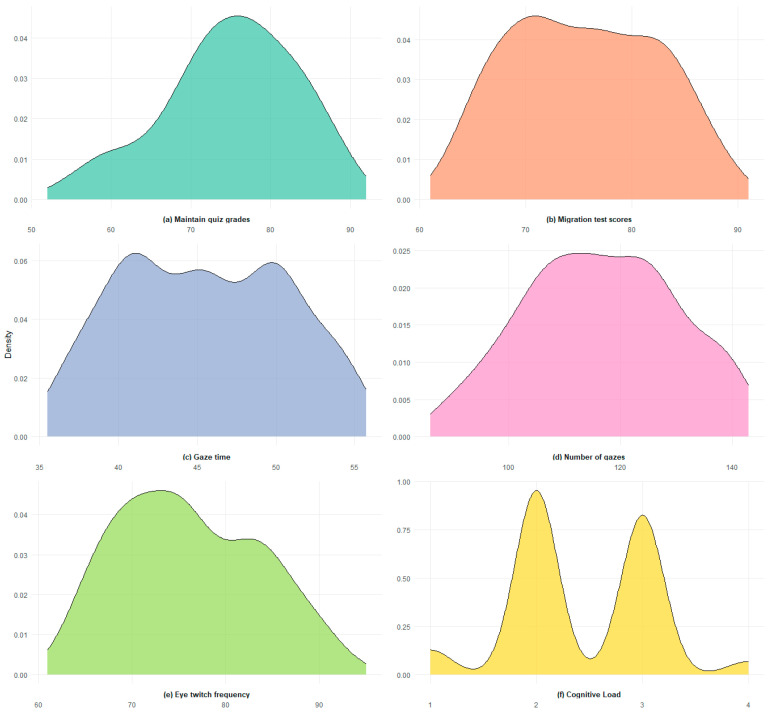
Probability density distribution plots.

Taken together, the 3D-interaction/intermediate-difficulty condition exhibited the most favorable performance across learning outcomes, eye-movement activity, and cognitive-load control, highlighting its combined advantages in supporting higher-order cognitive processes. In contrast, the conventional interaction condition performed less effectively at the intermediate difficulty level, suggesting potential limitations in more complex instructional contexts. As shown in Figure 3, probability density functions were used for visualization, with faceted layouts presenting the distributional characteristics of multiple variables. The kernel density estimation curves provided a smooth representation of each variable’s probability distribution, allowing readers to intuitively compare and interpret distributional patterns and central tendencies.

From the upper-right panel of Figure 3, it is evident that all variables included in the study approximate a normal distribution. As a non-continuous variable, cognitive load exhibited the highest frequencies at Levels 2 and 3, which served as mid-range categories, whereas Levels 1 and 4 occurred with lower frequencies as boundary categories. This overall distribution exhibited a pronounced central tendency and symmetry, which aligns with the fundamental characteristics of a normal distribution.

### 4.2. Two-Way ANOVA

Based on the ANOVA results presented in Table 2, the effects of interactive prompt type and task difficulty within the desktop virtual learning environment were summarized as follows.

A highly significant main effect of interactive prompt type was identified for both retention scores, F(1,228) = 163.715, *p* < 0.001, η^2^ = 0.395, MSE = 40.48, and transfer scores, F(1,228) = 944.070, *p* < 0.001, η^2^ = 0.679, MSE = 8.12, indicating that the 3D interactive condition yielded substantially higher learning outcomes than the conventional interactive condition. Significant interactions between interactive type and task difficulty were also observed for both measures (retention: F(1,228) = 22.530, *p* < 0.001, η^2^ = 0.054; transfer: F(1,228) = 215.940, *p* < 0.001, η^2^ = 0.155), suggesting that the relative effectiveness of the interaction types varied across difficulty levels. Task difficulty itself did not show a significant main effect (*p* > 0.05).

For the eye-tracking indicators, significant main effects of interactive prompt type were identified across all metrics (*p* < 0.001), with the largest effect found for fixation duration, F(1,228) = 968.840, *p* < 0.001, η^2^ = 0.704, MSE = 4.61. Task difficulty produced statistically significant yet modest effects on fixation count, F(1,228) = 4.37, *p* = 0.038, η^2^ = 0.006, MSE = 63.10, and saccade count, F(1,228) = 16.5, *p* < 0.001, η^2^ = 0.016, MSE = 12.90. Significant interactions between interactive type and difficulty were observed for all eye-movement metrics (*p* < 0.001), indicating that visual-attention patterns varied markedly across combinations of interaction modes and task difficulty.

Regarding cognitive load, a significant main effect of interactive prompt type was detected, F(1,228) = 327.085, *p* < 0.001, η^2^ = 0.527, MSE = 0.17, showing that the 3D interactive condition significantly lowered learners’ perceived cognitive load. Although task difficulty did not yield a significant main effect (*p* > 0.05), a significant interaction between interactive type and task difficulty was found, F(1,228) = 65.188, *p* < 0.001, η^2^ = 0.105, indicating that the impact of interactive prompts on cognitive load varied by difficulty level.

Overall, interactive prompt type was identified as the primary determinant of learning performance, eye-movement patterns, and cognitive load. The influence of task difficulty was comparatively limited; however, significant interactions across multiple dependent variables highlight the importance of jointly considering interaction type and content difficulty when examining learning processes.

### 4.3. Interaction Analysis

**Figure 4:**
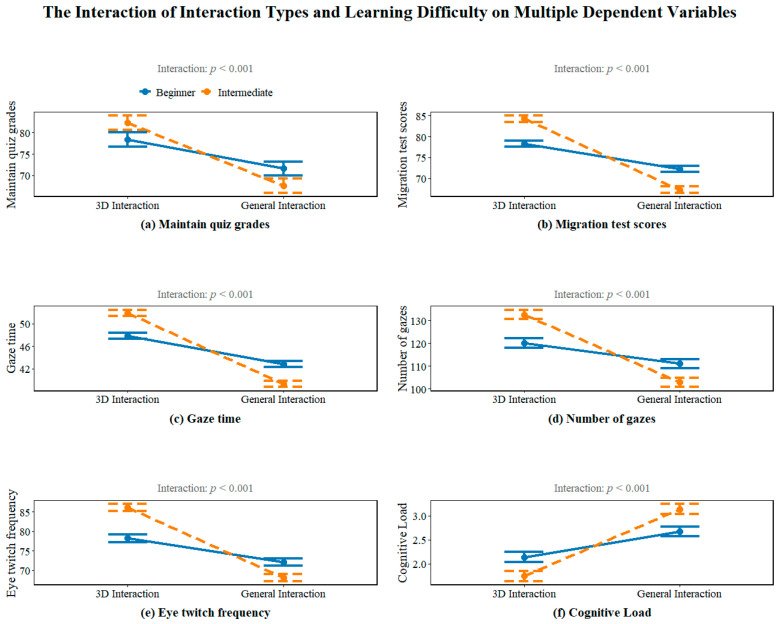
Interaction plot.

To illustrate the interaction effects between interactive prompt type and task difficulty more clearly, a series of interaction plots were generated. Figure 4 presents these visualizations for six key outcome variables: (a) retention test score, (b) transfer test score, (c) fixation duration, (d) fixation count, (e) saccade count, and (f) cognitive load level.

Figure 4 illustrates the interaction patterns between interactive prompt type and task difficulty across the six dependent variables, with all interaction effects achieving statistical significance (*p* < 0.001). Overall, the 3D interactive condition demonstrated distinct advantages over the conventional interaction condition across most measures, and these advantages were particularly pronounced at the intermediate difficulty level.

Specifically, for learning performance (Panels a,b), both retention and transfer scores were higher in the 3D interactive condition than in the conventional condition, with the most substantial differences observed at the intermediate level. The steeper slope in the 3D condition across difficulty levels suggests that 3D prompts provide greater instructional support as task complexity increases, whereas conventional prompts show limited scalability.

For eye-tracking indicators (Panels c–e), the 3D interactive condition yielded significantly longer fixation durations, higher fixation counts, and more frequent saccades, suggesting that learners allocated greater visual attention and cognitive resources in this condition. Notably, the increase in these metrics from introductory to intermediate difficulty was more pronounced in the 3D condition, indicating that 3D prompts may enhance attentional engagement particularly when processing complex content.

Regarding cognitive load (Panel f), lower load levels were consistently observed under the 3D interactive condition. This pattern aligns with the superior learning performance and heightened eye-movement activity observed in the same condition, suggesting that the enhanced learning outcomes associated with 3D interaction were not accompanied by an increase in cognitive burden. Instead, 3D prompts appear to support more efficient cognitive resource allocation.

Taken together, these findings reveal a robust interaction between interactive prompt type and task difficulty, with the 3D interactive condition producing the most optimal overall outcomes at the intermediate difficulty level. This evidence provides strong empirical support for advancing the design of interactive features in virtual learning environments, particularly for supporting learners in challenging instructional contexts.

### 4.4. Correlation Analysis

**Figure 5:**
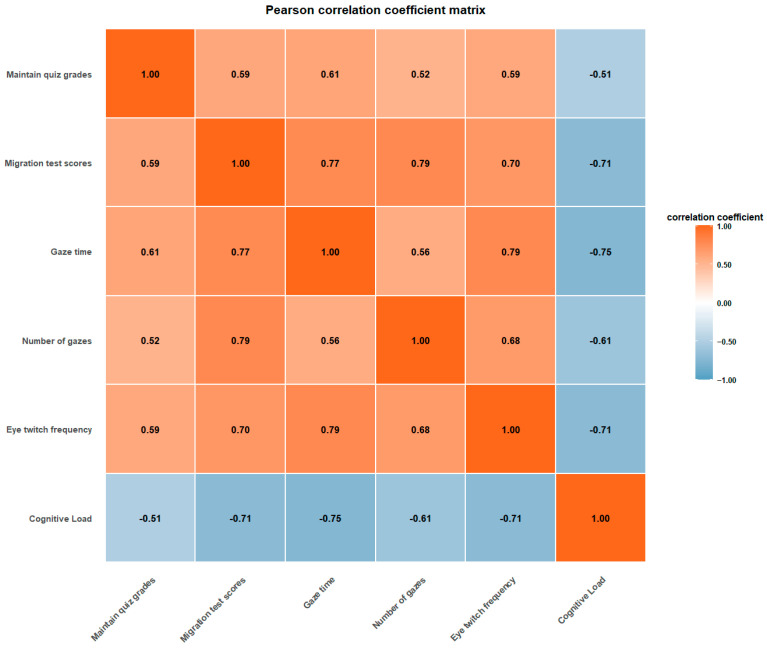
Heatmap of variable correlations.

To examine the underlying relational structure among the study variables, Pearson product–moment correlation coefficients were computed for six key variables: retention scores, transfer scores, fixation duration, fixation count, saccade frequency, and cognitive load. The correlation matrix was visualized using a heatmap, in which the color gradient ranged from blue (negative correlation) through white (zero correlation) to orange (positive correlation), thus allowing intuitive interpretation of both the magnitude and direction of linear relationships. The results are presented in Figure 5.

As shown in the Pearson correlation matrix in Figure 5, a systematic pattern of associations emerged across all six variables. Statistically significant linear correlations were identified for all variable pairs, with all absolute coefficients exceeding 0.50. With respect to academic performance, a moderate positive correlation was found between retention and transfer scores (r = 0.59), indicating that while both measures capture related aspects of learning outcomes, each represents distinct components of learning performance. Among the eye-tracking indicators, fixation duration and saccade frequency showed a strong positive correlation (r = 0.79), suggesting a close connection between visual-search activity and the time invested in information processing. Fixation count also displayed a strong positive correlation with transfer scores (r = 0.79), underscoring the relationship between visual-attention engagement and deeper learning outcomes.

Importantly, cognitive load was negatively correlated with all other variables, with the strongest associations observed for transfer scores (r = −0.71), fixation duration (r = −0.75), and saccade frequency (r = −0.71). These findings indicate that higher cognitive load substantially impedes learning performance and reduces learners’ investment in visual processing. More broadly, most eye-movement indicators exhibited moderate to strong positive associations with academic performance (r = 0.52–0.79), forming an interconnected cognitive-processing network, whereas cognitive load emerged as a key regulatory factor showing a consistent negative association with this network.

This correlation structure provides empirical support for the interdependence of learning performance, visual-attention investment, and cognitive load in virtual learning environments, reinforcing the relevance of cognitive resource-allocation theory within technology-enhanced learning contexts.

### 4.5. Heatmaps and Scan Paths of the Experimental Groups

**Figure 6:**
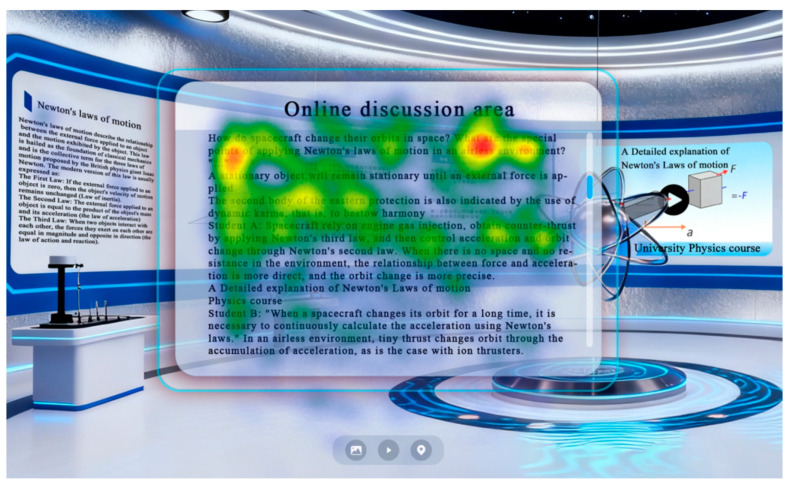
Visual-attention heatmap of the experimental groups.

**Figure 7:**
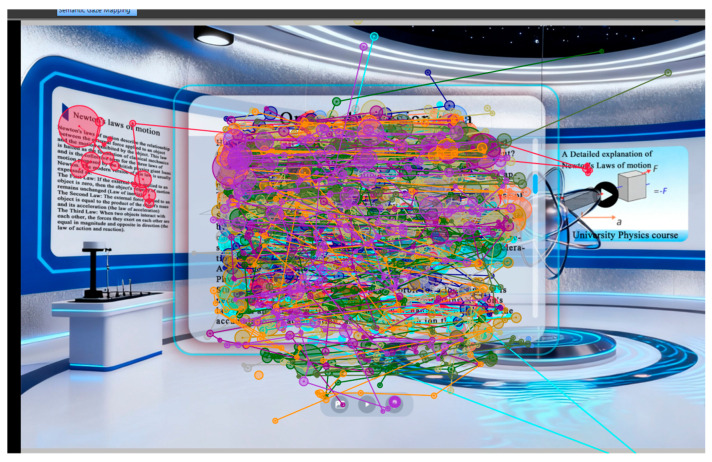
Scan-path visualization of the experimental groups.

To more clearly demonstrate the influence of 3D interactive prompts on learners’ visual attention within the desktop virtual learning environment, visual-attention heatmaps for the experimental groups are shown in Figure 6, and representative scan-path diagrams are displayed in Figure 7.

Heatmap visualizations (Figure 6) revealed that learners in the 3D interactive condition exhibited more concentrated fixations on key instructional elements (e.g., force vectors, pivot points) compared to the conventional condition, where attention was more dispersed across the interface. This suggests that 3D prompts effectively guided visual attention toward semantically relevant content, reducing visual search effort. Scan-path diagrams (Figure 7) further illustrated that learners exposed to 3D prompts demonstrated more systematic and efficient visual exploration patterns, with fewer regressive saccades and shorter scan-path lengths. These visual patterns align with the superior learning outcomes observed in the experimental group, supporting the hypothesis that 3D interactive prompts enhance attentional efficiency and cognitive integration.

## 5. Discussion

### 5.1. Application of Desktop Virtual Environments in University Physics

The findings of this study should be interpreted within the broader context of their application in university-level physics instruction. As widely accessible and low-cost instructional tools, desktop virtual environments derive their primary value from their ability to convert abstract, hazardous, or high-cost physical phenomena into interactive, exploratory dynamic models through advanced simulation and visualization. These environments facilitate the safe representation of extreme scenarios—including particle collisions and black-hole gravitational fields—and present otherwise intangible concepts such as Newtonian mechanics, electromagnetic fields, and quantum wave functions in an accessible and intuitive form. Such capabilities mitigate many of the conceptual challenges commonly encountered in theoretical physics learning, thereby enhancing learners’ conceptual understanding and their engagement in scientific inquiry. Within the quasi-experimental context of this study, the analytical results showed that 3D interactive prompts produced significant positive effects on both learning performance and eye-movement measures, confirming the effectiveness of desktop virtual environments as learning platforms.

Nevertheless, it must be acknowledged that the idealized models and procedural feedback inherent to virtual environments may constrain learners’ understanding of the inherent complexity of real-world physical systems and limit opportunities to develop hands-on problem-solving skills. Accordingly, desktop virtual environments should not be regarded as substitutes for physical experimentation but should instead function as critical supplements and enhancements to traditional instructional models. Future instructional practice should aim to construct an integrated learning cycle—comprising virtual preparation, theoretical elaboration, physical verification, and virtual extension— to fully capitalize on the advantages afforded by virtual simulation in overcoming cognitive barriers while preserving the irreplaceable role of physical experimentation in cultivating scientific literacy and experimental competence. This blended approach constitutes a critical trajectory in the modernization of physics education.

### 5.2. Dominant Role of Interaction Type and the Moderating Effect of Task Difficulty

The results indicated that interactive prompt type produced significant main effects across several dependent variables, particularly learning performance (retention and transfer scores) and eye-movement indicators (fixation duration, fixation count, and saccade frequency). The 3D interactive condition yielded the most favorable outcomes for intermediate-difficulty tasks, followed by introductory tasks; conversely, under the conventional interaction condition, performance was slightly higher for introductory than for intermediate tasks. These findings demonstrate that 3D interactive prompts provide more effective support for learning, with their advantages becoming especially pronounced when learners engage with more complex content. Interactive prompt type also had a significant effect on cognitive load, with consistently lower load levels observed under the 3D interactive condition—lowest for intermediate-difficulty tasks. This pattern reinforces the potential of 3D interactive prompts to alleviate cognitive burden and improve learning efficiency.

Although task difficulty alone did not produce significant main effects for most variables, its interaction with interactive prompt type was significant across several measures. Significant interaction effects were observed for fixation duration, fixation count, and saccade frequency, suggesting that task difficulty moderated the influence of interaction type. Learners achieved higher performance on intermediate-difficulty tasks when supported by 3D interactive prompts, whereas performance was superior for introductory tasks under conventional prompts. This cross-interaction pattern indicates that the effectiveness of interaction methods is not fixed but depends on the level of task difficulty. These findings provide important implications for instructional design and technology integration: in practical applications, the selection of interaction methods should be matched to the complexity of the learning content.

### 5.3. Attention-Guidance Mechanisms Revealed by Eye-Tracking Data

Eye-tracking evidence offered a fine-grained account of the mechanisms through which 3D interactive prompts shaped the learning process. The results indicated that 3D prompts significantly increased learners’ fixation duration and fixation count within key information regions, with effects especially pronounced during intermediate-difficulty tasks, in which fixation duration was substantially longer than in the conventional-interaction condition. These findings suggest that 3D prompts effectively captured learners’ initial attention and promoted deeper cognitive processing of core instructional content, aligning with prior research that emphasizes the role of attentional guidance in multimedia learning [7,9].

Heatmaps and scan-path visualizations further showed that visual attention in the 3D-prompt condition was more narrowly focused, with fewer ineffective saccades and reduced attentional dispersion, thereby demonstrating that the prompts effectively directed learners’ visual pathways and minimized unnecessary cognitive expenditure. This pattern is consistent with the signaling principle in multimedia learning, which posits that guiding attention to relevant elements enhances learning by reducing extraneous processing [21].

The strong positive association between fixation duration and learning performance offers further evidence for the role of “effective fixation” in supporting learning outcomes [17]. This relationship suggests that sustained attention on semantically relevant content facilitates schema construction and integration, a process that is particularly critical in complex, spatially rich learning environments such as DVLEs. Moreover, the correlation between saccade frequency and transfer scores implies that active visual exploration—supported by 3D prompts—may contribute to the development of flexible mental models that enable knowledge application in novel contexts.

Collectively, these findings demonstrate at the level of visual cognition that 3D interactive prompts optimize attentional allocation, promote efficient selection and integration of information, and ultimately enhance learning efficiency and deepen understanding. This aligns with cognitive load theory, which argues that well-designed instructional supports can manage working memory resources by directing attention toward essential information [26]. The eye-tracking data thus provide process-level validation for the effectiveness of 3D prompts in DVLEs, bridging the gap between instructional design principles and observable learning behaviors.

### 5.4. Limitations and Directions for Future Research

Despite the methodological rigor of this study, several limitations should be acknowledged. First, participants were recruited exclusively from first-year students at a single university. Although their disciplinary background provides a degree of representativeness, the homogeneity of the sample may constrain the generalizability of the findings. Second, the learning tasks were limited to physics mechanics. Although multiple difficulty levels were included, content from other scientific domains—such as biology or chemistry—was excluded; thus, the cross-disciplinary applicability of 3D interactive prompts has yet to be determined. Third, although eye-tracking indicators effectively captured patterns of visual attention, they were unable to capture deeper metacognitive strategies or emotional states that accompany cognitive processing. Finally, the study was conducted in a controlled laboratory environment, thereby leaving unexamined the long-term effects and adaptability of 3D prompts in authentic classroom or online learning settings.

Building on these findings and limitations, several avenues for future research are suggested. Broader samples and learning contexts warrant examination by recruiting learners from diverse regions, disciplines, and academic levels, and by undertaking longitudinal investigations in real instructional environments to assess the generalizability and sustained effects of 3D interactive prompts. Future work may also incorporate multimodal physiological data—such as EEG and heart-rate measures—together with qualitative learning analytics, including learning logs, to develop a more holistic characterization of the learning process and to elucidate the cognitive and affective pathways through which interactive prompts exert their influence. Additionally, the development of adaptive prompting systems informed by individual characteristics (e.g., prior knowledge, spatial ability) constitutes an important direction for achieving personalized instructional support. Expanding the application of 3D prompts to additional disciplinary domains, including chemistry and biology, and comparing their effectiveness across technological platforms such as VR and AR, may further refine strategies for integrating immersive technologies into education. Finally, systematically contrasting different prompt formats—such as dynamic annotations and voice-based guidance—may help clarify their differential effects on attention allocation and motivation, thereby contributing to more theoretically grounded and practically actionable design principles.

Although this study treated 3D interactive prompts as a unified intervention, future research should disentangle the effects of specific prompt types (e.g., dynamic annotations vs. animated demonstrations). It is plausible that annotations primarily support attentional guidance, whereas demonstrations facilitate mental model construction through procedural visualization.

## 6. Conclusions

The findings of this study indicate that, within desktop virtual learning environments, the use of 3D interactive prompts improves university students’ ability to learn moderately to highly complex physics concepts and concurrently reduces cognitive load. The importance of aligning interaction design with the nature of the learning content is further reinforced, suggesting that 3D interaction may offer distinct advantages when learners engage with tasks of greater complexity. Future research should examine how disciplinary contexts and individual learner characteristics—such as prior knowledge and spatial cognitive ability—moderate the effectiveness of different interaction types, thereby contributing to a more systematic understanding of how interaction design can be optimized in virtual learning environments.

## Figures and Tables

**Table 1:** Descriptive Statistics Results.

	Interaction Type	Learning Difficulty	Cases	Means	Median	Standard Deviation	Minimum	Maximum	Skewness	Kurtosis
Prior Knowledge	3D interaction	Intermediate	58	70.1	70	10.02	49	91	0.0522	0.618
		Introductory	58	71.8	72.5	9.22	51	88	−0.2298	−0.804
	Conventional interaction	Intermediate	58	71.8	72	7.66	53	86	−0.2768	−0.488
		Introductory	58	71.3	72	7.97	53	86	−0.2254	−0.498
Retention test score	3D interaction	Intermediate	58	82.28	83	5.17	72	92	−0.136	−0.817
		Introductory	58	78.34	79	5.975	66	89	−0.201	−0.862
	Conventional interaction	Intermediate	58	67.62	69	7.053	52	79	−0.497	−0.645
		Introductory	58	71.62	73	7.053	56	83	−0.497	−0.645
Transfer test score	3D interaction	Intermediate	58	84.26	84	2.85	78	91	0.190	−0.342
		Introductory	58	78.26	78	2.85	72	85	0.190	−0.342
	Conventional interaction	Intermediate	58	67.26	67	2.85	61	74	0.190	−0.342
		Introductory	58	72.26	72	2.85	66	79	0.190	−0.342
Fixation duration	3D interaction	Intermediate	58	51.89	51.92	2.186	47.90	55.70	−0.038	−1.260
		Introductory	58	47.88	47.86	2.177	43.90	51.70	−0.032	−1.245
	Conventional interaction	Intermediate	58	39.33	39.45	2.055	35.50	43.20	−0.060	−1.160
		Introductory	58	42.87	42.92	2.172	38.90	46.70	−0.031	−1.231
Fixation count	3D interaction	Intermediate	58	132.53	133	6.705	120	143	−0.133	−1.255
		Introductory	58	120.02	121	7.944	104	135	−0.176	−0.784
	Conventional interaction	Intermediate	58	102.83	104	8.45	86	119	−0.164	−0.703
		Introductory	58	110.98	112	8.544	94	127	−0.186	−0.756
Saccade count	3D interaction	Intermediate	58	86.02	85.5	3.527	79	95	0.326	−0.408
		Introductory	58	78.19	78	3.61	71	86	0.239	−0.720
	Conventional interaction	Intermediate	58	68.19	68	3.61	61	76	0.239	−0.720
		Introductory	58	72.19	72	3.61	65	80	0.239	−0.720
Cognitive load level	3D interaction	Intermediate	58	1.74	2	0.442	1	2	−1.132	−0.746
		Introductory	58	2.14	2	0.348	2	3	2.156	2.742
	Conventional interaction	Intermediate	58	3.14	3	0.348	3	4	2.156	2.742
		Introductory	58	2.67	3	0.473	2	3	−0.754	−1.483

**Table 2:** Analysis of variance results for the desktop virtual learning environment.

Variable	Fixed Factor	Sum of Squares	Degrees of Freedom		Mean Square		F Value	*p* Value	η^2^ Value
Prior Knowledge	Interaction type	21.1	1	21.1	0.275	0.601	0.001		
	Task difficulty	21.1	1	21.1	0.275	0.601	0.001		
	Interaction type × Task difficulty	62.1	1	62.1	0.807	0.370	0.004		
	Residual	17,540.2	228	76.90					
Retention score	Interaction type	6627.586	1		6627.586		163.715	<0.001	0.395
	Task difficulty	0.069	1		0.069		0.002	0.967	0.000
	Interaction type × Task difficulty	912.069	1		912.069		22.530	<0.001	0.054
	Residual	923.00	228		40.48				
Transfer score	Interaction type	7670.500	1		7670.500		944.070	<0.001	0.679
	Task difficulty	14.500	1		14.500		1.780	0.183	0.001
	Interaction type × Task difficulty	1754.500	1		1754.500		215.940	<0.001	0.155
	Residual	1852.50	228		8.12				
Fixation duration	Interaction type	4470.600	1		4470.600		968.840	<0.001	0.704
	Task difficulty	3.280	1		3.280		0.711	0.400	0.001
	Interaction type × Task difficulty	825.400	1		825.400		178.876	<0.001	0.130
	Residual	1052.08	228		4.61				
Fixation count	Task difficulty	21,763	1		21,763.00		344.80	<0.001	0.511
	Interaction type	276	1		275.90		4.37	0.038	0.006
	Interaction type × Task difficulty	6197	1		6196.60		98.18	<0.001	0.145
	Residual	14,391.00	228		63.10				
Saccade count	Interaction type	8232	1		8232.4		638.9	<0.001	0.614
	Task difficulty	212	1		212.4		16.5	<0.001	0.016
	Interaction type × Task difficulty	2028	1		2028.4		157.4	<0.001	0.151
	Residual	2938.00	228		12.90				
Load level	Task difficulty	54.069	1		54.069		327.085	<0.001	0.527
	Interaction type	0.069	1		0.069		0.417	0.519	0.001
	Interaction type × Task difficulty	10.7759	1		10.7759		65.188	<0.001	0.105
	Residual	37.69	228		0.17				

## Data Availability

Requests to access the data should be directed to the leading author.
